# Multiple Rare Risk Coding Variants in Postsynaptic Density-Related Genes Associated With Schizophrenia Susceptibility

**DOI:** 10.3389/fgene.2020.524258

**Published:** 2020-12-04

**Authors:** Tsung-Ming Hu, Ying-Chieh Wang, Chia-Liang Wu, Shih-Hsin Hsu, Hsin-Yao Tsai, Min-Chih Cheng

**Affiliations:** ^1^Department of Psychiatry, Yuli Branch, Taipei Veterans General Hospital, Hualien, Taiwan; ^2^Department of Future Studies and LOHAS Industry, Fo Guang University, Jiaosi, Taiwan; ^3^Institute of Medical Sciences, Tzu Chi University, Hualien City, Taiwan

**Keywords:** schizophrenia, PSD gene, resequencing, rare mutation, *in vitro*

## Abstract

**Objective:**

Schizophrenia is a chronic debilitating neurobiological disorder of aberrant synaptic connectivity and synaptogenesis. Postsynaptic density (PSD)–related proteins in *N*-methyl-D-aspartate receptor–postsynaptic signaling complexes are crucial to regulating the synaptic transmission and functions of various synaptic receptors. This study examined the role of PSD-related genes in susceptibility to schizophrenia.

**Methods:**

We resequenced 18 genes encoding the disks large-associated protein (DLGAP), HOMER, neuroligin (NLGN), neurexin, and SH3 and multiple ankyrin repeat domains (SHANK) protein families in 98 schizophrenic patients with family psychiatric history using semiconductor sequencing. We analyzed the protein function of the identified rare schizophrenia-associated mutants via immunoblotting and immunocytochemistry.

**Results:**

We identified 50 missense heterozygous mutations in 98 schizophrenic patients with family psychiatric history, and *in silico* analysis revealed some as damaging or pathological to the protein function. Ten missense mutations were absent from the dbSNP database, the gnomAD (non-neuro) dataset, and 1,517 healthy controls from Taiwan BioBank. Immunoblotting revealed eight missense mutants with altered protein expressions in cultured cells compared with the wild type.

**Conclusion:**

Our findings suggest that PSD-related genes, especially the NLGN, SHANK, and DLGAP families, harbor rare functional mutations that might alter protein expression in some patients with schizophrenia, supporting contributing rare coding variants into the genetic architecture of schizophrenia.

## Introduction

Schizophrenia is a chronic debilitating mental illness affecting approximately 1% of the global population and is characterized by overt psychotic (positive) symptoms, various deficits (negative symptoms), and impaired cognitive function (Freedman, 2003). On average, schizophrenia is estimated as approximately 80% hereditable, suggesting that genetic factors are involved in its pathogenesis (Mowry and Gratten, 2013). Studies have demonstrated a reduction of neuronal processes and synaptic dysfunction in schizophrenia (McGlashan and Hoffman, 2000; Hayashi-Takagi and Sawa, 2010), which is regulated by postsynaptic density (PSD)–related proteins in *N*-methyl-D-aspartate (NMDA) receptor–postsynaptic signaling complexes in various synaptic receptors (Takeuchi et al., 1997; Fukaya and Watanabe, 2000). Moreover, accumulating evidence from genetic, proteomic, and neuroanatomical studies indicates that aberrant synaptic plasticity and NMDA receptor–postsynaptic signaling complexes are involved in the pathophysiology of schizophrenia (Dracheva et al., 2001; Clinton and Meador-Woodruff, 2004; Kristiansen et al., 2007; Kirov et al., 2012; Focking et al., 2015). *In vitro* functional evidence together with human genetics data strongly suggests that mutations in a variety of postsynaptic scaffolding proteins may contribute to the etiology of schizophrenia (Sun et al., 2011; Peykov et al., 2015; Yu et al., 2018). Therefore, PSD-related genes involved in the formation and functional integrity of synapses can be considered candidate genes for schizophrenia.

In humans, the neuroligin (NLGN) gene family (*NLGN1*, *NLGN2*, *NLGN3*, *NLGN4X*, and *NLGN4Y*) encodes postsynaptic cell-adhesion molecules that are vital to the formation of functional synapses via trans-synaptic interactions with presynaptically expressed neurexin (NRXN) gene family and interactions with postsynaptic scaffolding molecules (Missler et al., 1998). Missense mutations in *NLGN2* or *NLGN3* have been associated with several neuropsychiatric diseases, such as schizophrenia (Sun et al., 2011) and autism (Jamain et al., 2003; Xu et al., 2014; Parente et al., 2017). The SH3 and multiple ankyrin repeat domains (SHANK) protein family comprises three main genes with highly similar domains that function as anchoring or scaffolding proteins to bridge postsynaptic membrane proteins and the cytoskeleton (Naisbitt et al., 1999; Tu et al., 1999). Mutations of the SHANK gene family (SHANK1-3) have been linked to psychiatric disorders such as schizophrenia, autism spectrum disorders, or intellectual disability (Wilson et al., 2003; Durand et al., 2007; Berkel et al., 2010; Sato et al., 2012; Peykov et al., 2015). The disks large-associated protein (DLGAP) family encoded by *DLGAP1*, *DLGAP2*, *DLGAP3*, and *DLGAP4* is involved in organizing the postsynaptic signaling complex in glutamatergic synapses and has been linked to a variety of psychiatric disorders (Takeuchi et al., 1997; Rasmussen et al., 2017). The homer protein family of dendritic proteins includes three members encoded by three different genes (*HOMER1*, *HOMER2*, and *HOMER3*) (Shiraishi-Yamaguchi and Furuichi, 2007). Homer proteins act as multimodal adaptors by interacting with several PSD proteins and are involved postsynaptically by regulating glutamatergic receptor trafficking, involving the function of plasma membrane ion channels and intracellular messenger systems (Thomas, 2002).

There has been growing debate that the genetic contribution to individual susceptibility to common complex diseases can be caused either by common disease-common variants with low penetrance or common disease-rare variants with high penetrance (Manolio et al., 2009; Schork et al., 2009; Sullivan et al., 2012). *De novo* mutations, including copy number variations and single-nucleotide variants (SNVs) of PSD-related proteins’ genes, have been identified in some sporadic patients with schizophrenia (Stefansson et al., 2008; Walsh et al., 2008; Xu et al., 2008, 2011; Grozeva et al., 2012; Fromer et al., 2014; Purcell et al., 2014; Xing et al., 2016). We previously identified several rare [minor allele frequency (MAF) < 0.01] schizophrenia-associated mutations in PSD-associated protein genes, including *DLGAP1*, *DLGAP2*, *DLGAP3*, and *NLGN2* (Sun et al., 2011; Li et al., 2013a, b; Li et al., 2014), which may contribute to some of the pathogenic mechanisms of schizophrenia in certain patients. Taken together, resequencing studies have implicated rare pathologic variants as susceptibility genes for schizophrenia.

To identify rare pathogenic SNVs, we resequenced 18 genes for PSD-related proteins, including members of the DLGAP (4 genes), HOMER (3 genes), NLGN (5 genes), NRXN (3 genes), and SHANK gene family (3 genes), in 98 schizophrenic patients with family psychiatric history using semiconductor sequencing technology. We analyzed the protein function of the identified rare schizophrenia-associated mutants via immunoblotting and immunocytochemistry.

## Materials and Methods

### Subjects and Study Outline

We recruited Han Chinese patients from Taiwan fulfilling the *Diagnostic and Statistical Manual of Mental Disorders, Fourth Edition* diagnostic criteria for schizophrenia. Healthy controls were from a medical center as usual medical check in east Taiwan. We excluded participants with organic brain syndromes, intellectual disability, substance-related psychosis, or mood disorder with psychotic features. The study was performed in two stages. At stage 1, 98 unrelated schizophrenic patients with family psychiatric history (46 males, mean age = 41 ± 9 years; 52 females, mean age = 45 ± 11 years) were screened for rare mutations using semiconductor sequencing. At stage 2, we genotyped the identified rare missense mutations and compared them with an independent sample of 484 nonrelated patients with schizophrenia (262 males, mean age = 48 ± 12 years; 222 females, mean age = 52 ± 10 years) and 533 healthy controls (252 males, mean age = 43 ± 15 years; 281 females, mean age = 45 ± 13 years) using Sanger sequencing.

This study was approved by Antai Medical Care Cooperation Antai-Tian-Sheng Memorial Hospital Institution Review Board (IRB no. 101020) and was performed in accordance with the 1964 World Medical Association’s Declaration of Helsinki and its later amendments. Written informed consent was obtained after the procedures are explained.

### Semiconductor Sequencing

Semiconductor sequencing was performed using the Ion Torrent^TM^ Personal Genome Machine^TM^ System (Thermo Fisher Scientific) according to the manufacturer’s protocol. Briefly, genomic DNA was isolated from peripheral blood cells of the subjects using a Gentra Puregene Blood Kit (QIAGEN) and quantified using the Qubit^TM^ dsDNA HS Assay Kit (Thermo Fisher Scientific). Custom amplification primers were designed using Ion AmpliSeq^TM^ Designer software (Thermo Fisher Scientific) to cover the exonic regions of 18 genes (*SHANK1*, *SHANK2*, *SHANK3*, *NRXN1*, *NRXN2*, *NRXN3*, *HOMER1*, *HOMER2*, *HOMER3*, *DLGAP1*, *DLGAP2*, *DLGAP3*, *DLGAP4*, *NLGN1*, *NLGN2*, *NLGN3*, *NLGN4X*, and *NLGN4Y*). Genomic DNA libraries were prepared using two primer pools and an Ion AmpliSeq^TM^ Library Kit 2.0 (Thermo Fisher Scientific). Each library was ligated to a different barcode adapter using an Ion Xpress^TM^ Barcode Adapters Kit (Thermo Fisher Scientific) and purified using Agencourt^TM^ AMPure^TM^ XP Reagent (Beckman Coulter). The concentrations of the purified libraries were determined by a quantitative polymerase chain reaction (PCR) with the Ion Library TaqMan^TM^ Quantitation Kit (Thermo Fisher Scientific). Emulsion PCR and subsequent Ion Sphere^TM^ particle enrichment were performed using the Ion PGM^TM^ Hi-Q View OT2 Kit and Ion PGM^TM^ Enrichment Beads on an Ion OneTouch^TM^ 2 system (Thermo Fisher Scientific). The final product was sequenced on the Ion 318^TM^ Chip using the Ion PGM^TM^ Hi-Q^TM^ View Sequencing Kit (Thermo Fisher Scientific). Data analysis, including alignment to the hg19 human reference genome, base calling, and variant calling, was performed using Torrent Suite built-in software. Variants were annotated using Ion Reporter^TM^ Software (Thermo Fisher Scientific).

### PCR Reaction and Fluorescence-Based Cycle Sequencing

PCR primer sequences were designed using the Primer3 website^1^. PCR amplification was performed according to our well-established laboratory protocols. For sequencing, aliquots of PCR products were purified using an Illustra^TM^ ExoProStar^TM^ 1-Step Kit (GE Healthcare Bio-Sciences) and then sequenced using a BigDye^TM^ Terminator v3.1 Cycle Sequencing Kit on a 3730 DNA Analyzer (Perkin Elmer Applied Biosystems) according to the manufacturer’s protocol.

### *In silico* Analysis

We explored whether the mutations we identified were documented in Taiwan BioBank^2^, a nationwide research database that collected, stored, and analyzed the biological data necessary for the research designed to trace the common chronic diseases occurring locally in Taiwan. A possible impact of amino acid substitution was analyzed using the publicly available prediction programs: Polyphen-2^3^, SIFT^4^, PMut^5^, and PROVEAN^6^. Evolutionary conservation was assessed using the multiz tool of the University of California Santa Cruz Genome Browser^7^.

### Construction of Expression Plasmids and Organelle Marker Vectors

A pcDNA3.1/C-terminal–green fluorescent protein (GFP) vector containing the coding sequence of human *NLGN2* (Sun et al., 2011) was used as a template to generate an NLGN2^*R*309*Q*^ mutant variant. Human *NLGN3*, *SHANK2*, *SHANK3*, *DLGAP1*, and *DLGAP3* cDNAs were cloned into expression vectors (tGFP and Myc-DDK tagged) using the PrecisionShuttle vector System (OriGene). Vectors containing the mutant were generated using a QuikChange^®^ Lightning Site-Directed Mutagenesis kit (Agilent Technologies). The authenticity of the cloned sequences was confirmed by qualitative restriction enzyme digestion and Sanger sequencing. Human lymphocyte-specific protein tyrosine kinase (LCK) with a tag vector for plasma membrane marking was purchased from OriGene.

### Cell Culture, Transfection, and Immunocytochemistry

HEK-293 and neuro-2a cells were cultured in minimal essential media supplemented with 1 mM sodium pyruvate, 0.1 mM nonessential amino acids, 100 U penicillin, 100 μg streptomycin (Invitrogen), and 10% fetal bovine serum. The cells were cultured on 24-well plates (10,000 cells/well) and cotransfected with 450 ng of reporter plasmid and 50 ng of organelle marker vector using Lipofectamine^TM^ 3000 (Invitrogen). After 24 h, the cells were washed with cold phosphate-buffered saline (PBS) and fixed in 4% paraformaldehyde in PBS (pH 7.4) for 30 min at 37°C. After that, they were washed three times with PBS and permeabilized for 15 min in PBS containing 0.1% Triton X-100. The fixed and permeabilized cells were simultaneously stained with Alexa Fluor^®^ 594 wheat germ agglutinin (WGA) (Invitrogen) and mounted in a medium containing 4′,6-diamidino-2-phenylindole (VECTOR Laboratories). Images were acquired using a fluorescence microscope and analyzed with ZEN 2 software (Zeiss).

### Protein Preparation and Immunoblotting Analysis

HEK-293 and neuro-2a cells were transfected in 6-well plates. After 24 h of expression, they were washed twice with cold PBS and resuspended in lysis solution containing 20 mM HEPES (pH 7.6), 7.5 mM NaCl, 2.5 mM MgCl_2_, 0.1 mM EDTA, 0.1% Triton X-100, 0.1 mM Na_3_VO_4_, and 50 mM NaF, and protease inhibitor cocktail tablet (one complete mini tablet/10 mL; Roche Diagnostics GmbH). The homogenates were centrifuged at 13,000 rpm for 30 min at 4°C, and the supernatants were stored at −20°C until use. Membrane proteins were extracted using a Subcellular Protein Fractionation Kit for Cultured Cells (Thermo Fisher Scientific) according to the manufacturer’s protocol.

Immunoblotting was performed according to our standard protocol using primary antibodies (anti-NLGN2 and anti-GFP, Santa Cruz Biotechnology; anti-SHANK3, anti-tGFP, and anti-DDK, OriGene) and secondary antibodies (anti-mouse, Kirkegaard and Perry Laboratories; anti-rabbit, GE Healthcare; anti-goat, Santa Cruz Biotechnology). The chemiluminescence signal was visualized using a Trident ECL plus kit (GeneTex). Band intensities were assessed utilizing the National Institutes of Health ImageJ software^8^ and normalized to the intensity of the GFP or tGFP band.

### Statistical Analysis

In the immunoblotting study, we used the Mann-Whitney *U*-test to compare the means between two groups (wild and mutant types) and analyze statistical significance. The Kruskal–Wallis *H*-test was compared among three or more independent groups. *p* < 0.05 was considered statistically significant. All the calculations were implemented using SPSS 18th version.

## Results

### Identification of Variants of PSD-Related Genes in Schizophrenia

Our custom-designed panel covering the exon regions of 18 genes allowed the analysis of 267 exons (5-bp exon padding) by the targeted resequencing of 755 amplicons (96.72% coverage). We initially screened for mutations in 98 schizophrenic patients with family psychiatric history. Data generation from semiconductor sequencing was listed in Supplementary Table 1. After the semiconductor sequencing of all the amplicons, 50 heterozygous missense mutations were identified (Table 1). The genotypes of these 50 missense mutations in 98 patients with schizophrenia are listed in Supplementary Table 2. The genotype frequency distributions of the 49 mutants did not deviate from Hardy–Weinberg equilibrium in the patient group, but the genotype distribution of DLGAP2^*P*384*Q*^ did significantly deviate from Hardy–Weinberg equilibrium. Five missense mutations (DLGAP2^*P*384*Q*^, NRXN2^*L*81*Q*^, SHANK1^*D*897*E*^, SHANK1^*V*1504*A*^, and DLGAP4^*R*747*Q*^) with MAF greater than 5% were identified. Ten missense mutations (DLGAP3^*H*22*N*^, DLGAP3^*G*352*E*^, DLGAP3^*S*455*R*^, NRXN2^*P*642*T*^, SHANK2^*L*158*F*^, NLGN2^*R*309*Q*^, DLGAP1^*F*70*L*^, SHANK3^*P*209*A*^, NLGN3^*V*346*M*^, and NLGN3^*Q*691*R*^) were absent from the dbSNP database, the gnomAD (non-neuro) dataset, and 1,517 healthy controls from Taiwan BioBank, indicating that they were rare mutations. One patient harbored two rare missense mutations (DLGAP1^*F*70*L*^/NLGN3^*Q*691*R*^), and another a double-missense mutation (NLGN3^*R*195*Q*/*V*346*M*^) in the *NLGN3* gene.

**TABLE 1:** Fifty missense mutations identified from 98 patients with schizophrenia in the semiconductor sequencing stage.

Genomic position	Gene symbol	Variant	Amino acid change	dbSNP ID	gnomAD (non-neuro) MAF	Taiwan Biobank MAF	*In silico* analysis for amino acid substitution			
	
							Polyphen-2	SIFT	Pmut	Provean
chr1:35370921	DLGAP3	c.64C > A	p.H22N	N/R	N/R	N/R	Benign	Tolerated	Neutral	Neutral
chr1:35370851 chr1:35370852	DLGAP3	c.133_134 delCCinsAA	p.P45N	rs552097335	0.0002792	0.004642	Possibly damaging	Tolerated	Pathological	Neutral
				rs567109248	0.0002817	0.004707				
chr1:35370693	DLGAP3	c.292C > A	p.P98T	rs201890156	0.001111	0.013937	Possibly damaging	Tolerated	Neutral	Neutral
chr1:35370518	DLGAP3	c.467C > T	p.T156M	rs144823481	0.0001194	N/R	Probably damaging	Affect protein function	Neutral	Neutral
chr1:35369930	DLGAP3	c.1055G > A	p.G352E	N/R	N/R	N/R	Possibly damaging	Tolerated	Pathological	Neutral
chr1:35365293	DLGAP3	c.1363A > C	p.S455R	N/R	N/R	N/R	Benign	Tolerated	Pathological	Neutral
chr2:50318584	NRXN1	c.3595G > A	p.A1199T	rs201336161	0.0003652	0.006592	Benign	Tolerated	Pathological	Neutral
chr2:50280526	NRXN1	c.3921G > C	p.E1307D	rs200935246	0.0001346	0.003955	Benign	Tolerated	Neutral	Neutral
chr2:50280477	NRXN1	c.3970A > T	p.T1324S	rs202006815	0.0001923	0.004944	Benign	Tolerated	Neutral	Neutral
chr8:1514009	DLGAP2	c.1151C > A	p.P384Q	rs2301963	0.4904	0.466359	Possibly damaging	Tolerated	Pathological	Neutral
chr8:1581158	DLGAP2	c.1516T > C	p.C506R	rs753092749	0.00002911	0.001318	Possibly damaging	Affect protein function	Pathological	Deleterious
chr8:1626621	DLGAP2	c.2290C > G	p.P764A	rs372823337	0.00004419	0.000990	Benign	Tolerated	Neutral	Deleterious
chr8:1649394	DLGAP2	c.2750C > T	p.P917L	rs1562601334	0.00004410	0.000660	Benign	Tolerated	Pathological	Neutral
chr11:64480930	NRXN2	c.242T > A	p.L81Q	rs12273892	0.1293	0.188596	Possibly damaging	Tolerated	Neutral	Neutral
chr11:64435078	NRXN2	c.1442A > T	p.D481V	rs376239889	0.0001442	0.003630	Benign	Tolerated	Neutral	Deleterious
chr11:64428486	NRXN2	c.1924C > A	p.P642T	N/R	N/R	N/R	Probably damaging	Affect protein function	Neutral	Deleterious
chr11:70858365	SHANK2	c.8G > A	p.R3H	rs369450251	0.0002780	0.002307	Probably damaging	Affect protein function	Neutral	Neutral
chr11:70830074	SHANK2	c.212G > A	p.C71Y	rs182549877	0.00006156	0.003955	Probably damaging	Affect protein function	Pathological	Deleterious
chr11:70829901	SHANK2	c.385G > A	p.V129M	rs73521173	0.01453	0.029334	Benign	Tolerated	Neutral	Neutral
chr11:70824391	SHANK2	c.431T > C	p.V144A	rs782270817	0.00009206	0.005274	Probably damaging	Affect protein function	Neutral	Deleterious
chr11:70824350	SHNAK2	c.472C > T	p.L158F	N/R	N/R	N/R	Probably damaging	Affect protein function	Neutral	Deleterious
chr11:70666725	SHANK2	c.1237A > G	p.A413T	rs782699631	0.00009662	N/R	Benign	Affect protein function	Neutral	Neutral
chr11:70666634	SHANK2	c.1328G > A	p.R443H	rs782229916	0.00006215	0.000688	Probably damaging	Affect protein function	Neutral	Neutral
chr11:70653166	SHANK2	c.1604A > G	p.K535R	rs182586961	0.0002768	0.007591	Possibly damaging	Tolerated	Neutral	Neutral
chr11:70333379	SHANK2	c.3019G > A	p.A1007T	rs781910453	0.000009614	N/R	Probably damaging	Tolerated	Neutral	Neutral
chr14:79434660	NRXN3	c.1994G > A	p.R665Q	rs151072919	0.0001057	0.001318	Benign	Tolerated	Pathological	Neutral
chr14:80328263	NRXN3	c.3142G > A	p.G1048S	rs200533333	0.0001835	0.000989	Benign	Tolerated	Neutral	Neutral
chr15:83519953	HOMER2	c.826A > G	p.I276V	rs373795200	0.0002328	0.004944	Benign	Tolerated	Neutral	Neutral
chr17:7318356	NLGN2	c.926G > A	p.R309Q	N/R	N/R	N/R	Possibly damaging	Tolerated	Neutral	Neutral
chr17:7320472	NLGN2	c.1862G > A	p.R621H	rs756129752	0.00005748	N/R	Probably damaging	Affect protein function	Neutral	Neutral
chr18:3879859	DLGAP1	c.210C > A	p.F70L	N/R	N/R	N/R	Probably damaging	Affect protein function	Pathological	Deleterious
chr18:3581916	DLGAP1	c.1922A > G	p.K641R	rs764287181	0.0001697	0.002637	Benign	Tolerated	Neutral	Neutral
chr19:51207043	SHANK1	c.1267C > G	p.P423A	rs552906733	0.00007192	0.000491	Benign	Tolerated	Neutral	Deleterious
chr19:51172526	SHANK1	c.2691C > G	p.D897E	rs41275782	0.05668	0.111551	Benign	Tolerated	Neutral	Neutral
chr19:51170706	SHANK1	c.4511T > C	p.V1504A	rs3745521	0.2854	0.436475	Benign	Tolerated	Neutral	Neutral
chr19:51165932	SHANK1	c.5776G > A	p.D1926N	rs374230001	0.00009214	0.000439	Benign	Affect protein function	Neutral	Neutral
chr19:51165505	SHANK1	c.6203C > G	p.A2068G	rs567977286	0.0001911	0.003038	Probably damaging	Affect protein function	Pathological	Neutral
chr19:51165488	SHANK1	c.6220C > T	p.R2074C	rs549670535	0.00008803	N/R	Possibly damaging	Affect protein function	Pathological	Deleterious
chr20:35075310	DLGAP4	c.1618G > A	p.G540S	rs142893536	0.001153	0.001009	Benign	Tolerated	Neutral	Neutral
chr20:35128751	DLGAP4	c.2240G > A	p.R747Q	rs41274714	0.02724	0.069036	Probably damaging	Tolerated	Neutral	Neutral
chr22:51117471	SHANK3	c.625C > G	p.P209A	N/R	N/R	N/R	Possibly damaging	Affect protein function	Neutral	Deleterious
chr22:51117580	SHANK3	c.734T > C	p.I245T	rs9616915	0.4215	0.069490	Benign	Tolerated	Neutral	Neutral
chr22:51159778	SHANK3	c.3517G > A	p.A1173T	rs139686326	0.0009222	0.008262	Benign	Tolerated	Neutral	Neutral
chr22:51160049	SHANK3	c.3788C > T	p.P1263L	rs757572910	0.0001648	0.003640	Probably damaging	Tolerated	Pathological	Deleterious
chrX:70375070	NLGN3	c.584G > A	p.R195Q	rs190164205	0.00005276	0.000688	Benign	Affect protein function	Pathological	Neutral
chrX:70386983	NLGN3	c.1036G > A	p.V346M	N/R	N/R	N/R	Possibly damaging	Tolerated	Neutral	Neutral
chrX:70389472	NLGN3	c.2072A > G	p.Q691R	N/R	N/R	N/R	Benign	Tolerated	Neutral	Neutral
chrX:6069437	NLGN4X	c.71A > G	p.N24S	rs775784070	0.00004609	N/R	Benign	Tolerated	Neutral	Neutral
chrX:6069116	NLGN4X	c.392A > G	p.N131S	rs145307351	0.0003884	0.001345	Benign	Tolerated	Neutral	Deleterious
chrY:16734196	NLGN4Y	c.197T > C	p.I66T	rs749178206	0.001139	0.009576	Benign	Tolerated	Neutral	Neutral

*Reference version, GRCh37; DLGAP1, NM_004746.3; DLGAP2, NM_004745.4; DLGAP3, NM_001080418.2; DLGAP4, NM_014902.5; HOMER2, NM_199330.2; NLGN2, NM_020795.3; NLGN3, NM_181303.1; NLGN4X, NM_181332.2; NLGN4Y, NM_014893.4; NRXN1, NM_004801.5; NRXN2, NM_015080.3; NRXN3, NM_004796.5; SHANK1, NM_016148.3; SHANK2, NM_012309.4; SHANK3, NM_033517.1; N/R, not registered.*

**FIGURE 1:**
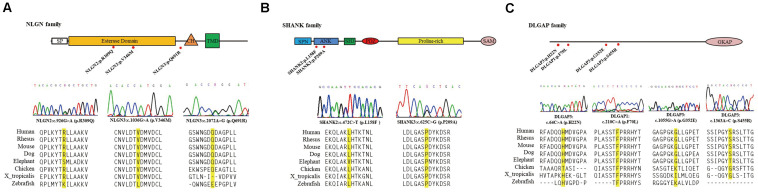
Schematic genomic structures of the NLGN **(A)**, SHANK **(B)**, and DLGAP **(C)** gene families. The locations and sequence electropherograms of the variants found in patients with schizophrenia are shown. The sequence alignment of eight mammalian species indicates the conservation of the mutated amino acids. SP, signal peptide domain; CH, cholinesterase domain; TMD, transmembrane domain; SPN, shank/ProSAP N-terminal domain; ANK, ankyrin repeats domain; SH3, SRC homology 3 domain; PDZ, PSD-95/D/ZO-1 domain; SAM, sterile α motif; GKAP, guanylate kinase-associated protein.

Next, we focused on ten missense variants absent from the gnomAD (non-neuro) dataset and Taiwan BioBank database for a genotyping assay in an independent sample set to ascertain whether these mutations were patient-associated in our Taiwan sample. These 10 missense mutations were also absent from a new collection of 468 patients with schizophrenia and 533 healthy controls from Taiwan (Supplementary Table 3). NRXN2^*P*642*T*^ is located at presynaptic NRXNs, which form a transsynaptic complex with postsynaptic NLGNs (Boucard et al., 2005). We focused on the 9 rare missense mutations (DLGAP3^*H*22*N*^, DLGAP3^*G*352*E*^, DLGAP3^*S*455*R*^, SHANK2^*L*158*F*^, NLGN2^*R*309*Q*^, DLGAP1^*F*70*L*^, SHANK3^*P*209*A*^, NLGN3^*V*346*M*^, and NLGN3^*Q*691*R*^) located at postsynaptic regions for further *in silico* analysis and functional assays. The schematic genomic structures of the SHANK, DLGAP, and NLGN gene families and the positions of these 9 patient-associated missense mutations are illustrated in Figure 1. Evolutionary conservation analysis revealed that the amino acids corresponding to the nine rare mutations were highly conserved among eight species (Figure 1). *In silico* analysis showed that seven mutations (DLGAP3^*G*352*E*^, DLGAP3^*S*455*R*^, SHANK2^*L*158*F*^, NLGN2^*R*309*Q*^, SHANK3^*P*209*A*^, NLGN3^*V*346*M*^, and NLGN3^*Q*691*R*^) were predicted to be pathogenic in at least one prediction programs, whereas DLGAP1^*F*70*L*^ was predicted to be pathogenic in all four prediction programs (Table 1).

### Immunoblotting Analysis of Gene Mutants in Cultured Cells

**FIGURE 2:**
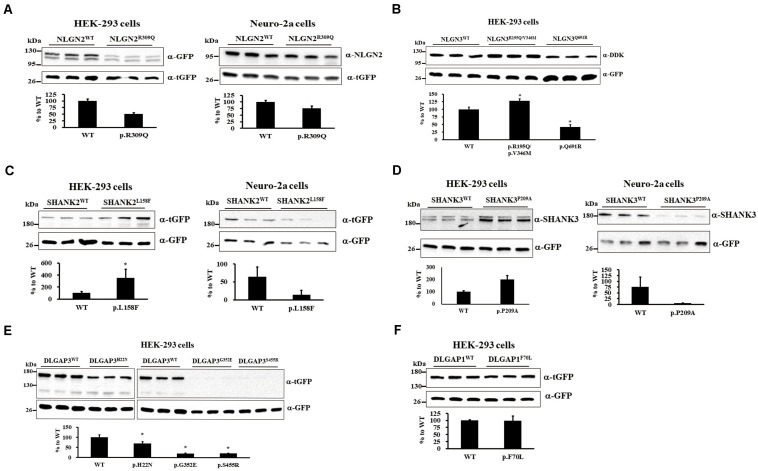
Immunoblotting of gene mutants in cultured cells. **(A)** Immunoblotting of protein extracts from HEK-293 cells (left) and neuro-2a cells (right) transiently cotransfected with a plasmid expressing either NLGN2^*WT*^ or NLGN2^*R*309*Q*^ cDNAs with pCMV6-AC-tGFP. **(B)** Immunoblotting of protein extracts from HEK-293 cells transiently cotransfected with a plasmid expressing NLGN3^*WT*^, NLGN3^*R*195*Q*/*V*346*M*^, or NLGN3^*Q*691*R*^ cDNAs with pcDNA3.1-GFP. **(C)** Immunoblotting of protein extracts from HEK-293 cells (left) and neuro-2a cells (right) transiently cotransfected with a plasmid expressing either SHANK2^*WT*^ or SHANK2^*L*158*F*^ cDNAs with pcDNA3.1-GFP. **(D)** Immunoblotting of protein extracts from HEK-293 cells (left) and neuro-2a cells (right) transiently cotransfected with a plasmid expressing either SHANK3^*WT*^ or SHANK3^*P*209*A*^ cDNAs with pcDNA3.1-GFP. **(E)** Immunoblotting of protein extracts from HEK-293 cells transiently cotransfected with a plasmid expressing DLGAP3^*WT*^ or DLGAP3 mutant cDNAs (DLGAP3^*H*22*N*^, DLGAP3^*G*352*E*^, and DLGAP3^*S*455*R*^) with pcDNA3.1-GFP. **(F)** Immunoblotting of protein extracts from HEK-293 cells transiently cotransfected with a plasmid expressing either DLGAP1^*WT*^ or DLGAP1^*F*70*L*^ cDNAs with pCMV6-AC-GFP. For normalization, lysates were analyzed in parallel by anti-tGFP or anti-GFP immunoblotting. Results are representative of three independent experiments. WT, wild-type. Graphs represent means ± standard errors. *p < 0.05.

We investigated whether 9 rare missense mutations affected protein expression via immunoblotting of HEK-293 or neuro-2a cells after 24 h of transiently expressing wild-type (WT) or mutant protein. NLGN2-GFP fusion protein expression tended to reduce in HEK-293 and neuro-2a cells carrying the NLGN2^*R*309*Q*^ mutant compared with NLGN2^*WT*^ (Figure 2A). The Mann–Whitney *U*-test was performed between the two conditions each time (HEK-293, *p* = 0.1; neuro-2a, *p* = 0.1). NLGN3-DDK fusion protein expression was significantly increased in HEK-293 cells carrying the NLGN3^*R*195*Q*/*V*346*M*^ mutant, whereas it was significantly decreased in cells carrying the NLGN3^*Q*691*R*^ mutant compared with NLGN3^*WT*^ (Figure 2B). A Kruskal–Wallis *H*-test was performed between the three conditions (*p* = 0.027). The SHANK2-tGFP fusion protein with the SHANK2^*L*158*F*^ mutant was significantly increased in HEK-293 cells but tended to decrease in neuro-2a cells compared with SHANK2^*WT*^ (Figure 2C). The Mann–Whitney *U*-test was performed between the two conditions (HEK-293, *p* = 0.002; neuro-2a, *p* = 0.1). The protein expression of the SHANK3-tGFP fusion protein with SHANK3^*P*209*A*^ tended to increase in HEK-293 cells but tended to decrease in neuro-2a cells compared with SHANK3^*WT*^ (Figure 2D). The Mann–Whitney *U*-test was performed between the two conditions each time (HEK-293, *p* = 0.1; neuro-2a, *p* = 0.1). Immunoblotting of cell lysates from the HEK-293 cells carrying DLGAP3^*WT*^ and DLGAP3^*H*22*N*^, DLGAP3^*G*352*E*^, and DLGAP3^*S*455*R*^ revealed significantly decreased levels of DLGAP3-tGFP fusion proteins in mutants relative to WT cells (Figure 2E). A Kruskal–Wallis *H*-test was performed between the four conditions (*p* = 0.034). HEK-293 cells with DLGAP1^*F*70*L*^ mutant demonstrated similar levels of DLGAP1-tGFP fusion protein expression as the WT cells (Figure 2F). The Mann–Whitney *U*-test was performed between the two conditions each time (*p* = 0.7).

### Localization Analysis of Gene Mutants in Cultured Cells

**FIGURE 3:**
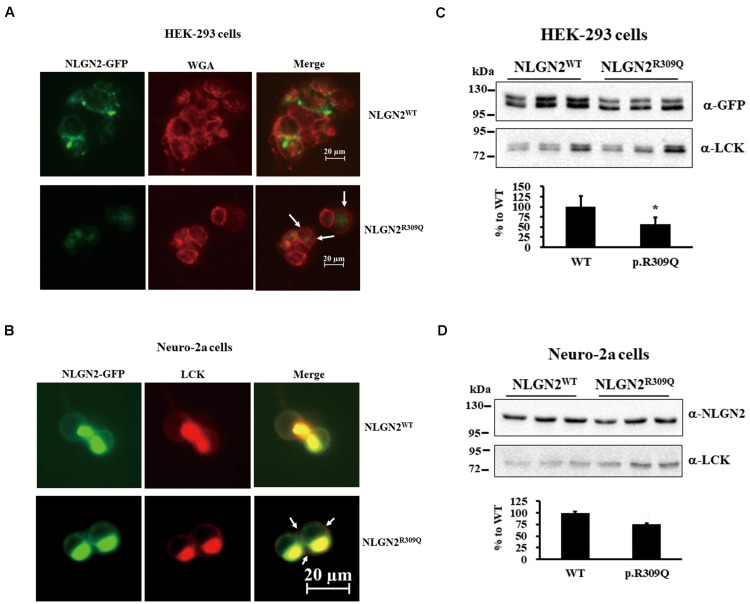
NLGN2-GFP fusion protein expression was reduced in the membrane fraction of cultured cells carrying NLGN2^*R*309*Q*^ mutant compared with the WT construct. **(A)** Subcellular localization of NLGN2 fusion GFP protein in HEK-293 cells transiently cotransfected either with NLGN2^*WT*^ or NLGN2^*R*309*Q*^ plasmids. WT proteins colocalized to the membrane in transfected HEK-293 cells, whereas NLGN2^*R*309*Q*^ mutant proteins colocalized to the membrane (WGA) at a lower level (arrow). **(B)** Subcellular localization of NLGN2 fusion GFP protein in neuro-2a cells transiently cotransfected either with NLGN2^*WT*^ or NLGN2^*R*309*Q*^ plasmids and LCK plasmids. WT proteins colocalized to the plasma membrane marker (LCK) in transfected neuro-2a cells, whereas NLGN2^*R*309*Q*^ mutant proteins colocalized to LCK at a lower level (arrow). **(C)** Immunoblotting of protein extracts from the membrane fraction of HEK-293 overexpressed either with NLGN2^*WT*^ or NLGN2^*R*309*Q*^ plasmid. **(D)** Immunoblotting of protein extracts from the membrane fraction of neuro-2a cells overexpressed either with NLGN2^*WT*^ or NLGN2^*R*309*Q*^ plasmid. WT, wild-type. WGA and LCK, membrane protein markers wheat germ agglutinin and lymphocyte-specific protein tyrosine kinase, respectively. Graphs represent means ± standard errors. *p < 0.05.

To understand the effects of rare mutations on protein translocation, we examined the protein subcellular localization of the WT and the mutant proteins in HEK-293 and neuro-2a cells. GFP signal was observed in the membrane fraction (WGA) of HEK-293 transfected with NLGN2^*WT*^-GFP plasmid but not NLGN2^*R*309*Q*^-GFP plasmid (Figure 3A). NLGN2 WT proteins colocalized to the plasma membrane marker (LCK) in transfected neuro-2a cells, whereas NLGN2^*R*309*Q*^ mutant proteins colocalized to LCK at a lower level (Figure 3B). We further examined and quantified the presence of the overexpressed NLGN2-GFP fusion protein in the membrane fraction using an immunoblot assay. The NLGN2-GFP fusion protein was significantly reduced in the membrane fraction of HEK-293 cells carrying NLGN2^*R*309*Q*^ mutant but tended to reduce in neuro-2a cells carrying NLGN2^*R*309*Q*^ mutant compared with the WT construct (Figures 3C,D). The Mann–Whitney *U*-test was performed between the two conditions each time (HEK-293, *p* = 0.015; neuro-2a, *p* = 0.1). Immunocytochemistry revealed NLGN3^*WT*^, NLGN3^*R*195*Q*/*V*346*M*^, and NLGN3^*Q*691*R*^ proteins colocalized to LCK, whereas SHANK2^*WT*^, SHANK2^*L*158*F*^, SHANK3^*WT*^, SHANK3^*P*209*A*^, DLGAP3^*WT*^, DLGAP3^*H*22*N*^, DLGAP3^*G*352*E*^, and DLGAP3^*S*455*R*^ mostly localized in the cytosol (Supplementary Figure 1).

### Clinical Findings of the Patients With Rare Missense Mutations

**FIGURE 4:**
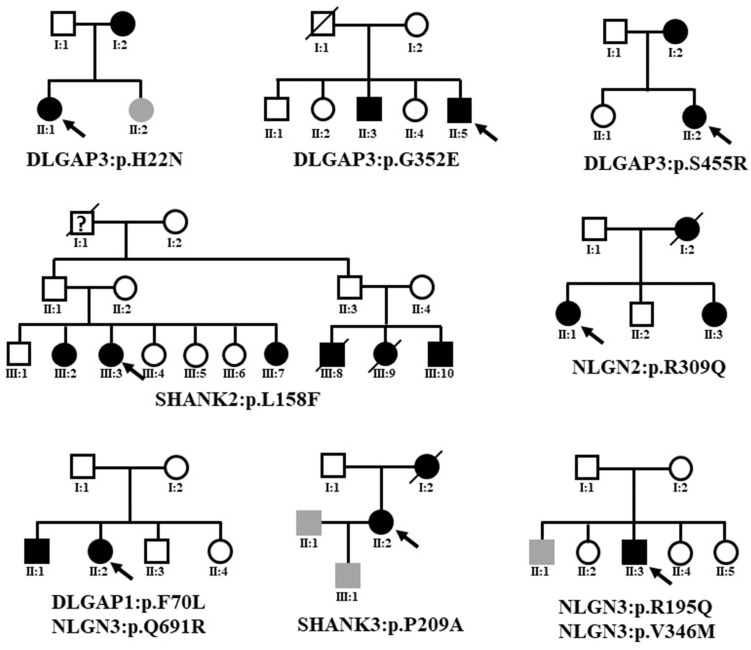
Familial relationships of the patients with schizophrenia with rare coding variants identified in this study. The black symbol indicates schizophrenia. The gray symbol indicates an intellectual disability. The question mark indicates unclear psychosis. Black arrows indicate the index case subjects.

Because eight variants that altered protein expression were absent from the dbSNP database, the gnomAD (non-neuro) dataset, and Taiwan BioBank, we presumed that they existed in the multiple psychosis family. The familial relationships of the patients with schizophrenia with rare coding variants identified in this study are presented in Figure 4. Details regarding patient history are shown in Supplementary Table 4. We did not perform segregation analysis in multiplex pedigrees; we then could not confirm that the identified rare missense mutations were *de novo* or transmitted from parents to multiple affected members.

## Discussion

Schizophrenia is a disorder involving polygenic inheritance, with rare variants having large effects on risk and common variants having minor impacts (Craddock et al., 2007; McClellan et al., 2007). A genome-wide association study (GWAS) has been used to evaluate an association with common genetic variants or markers (typically with an MAF > 5 %) with schizophrenia (Schizophrenia Working Group of the Psychiatric Genomics Consrotium, 2014). Because GWAS focuses on identifying common variants, it is plausible that low frequency (MAF < 5%) could explain additional trait variability of schizophrenia. Accumulated studies demonstrated that loss-of-function variants and missense variants were enriched in genes with shared similar cellular function, having clinical implications for finding additional drug targets (Lips et al., 2012; Rees et al., 2019). Targeted sequencing focuses on many targeted regions, such as a functional gene group, and identifies sequence variants with high confidence and accuracy (Bewicke-Copley et al., 2019). PSD proteins are essential for protein trafficking in neurons and synaptic plasticity, processes commonly associated with schizophrenia pathogenesis (Pocklington et al., 2014). Here, to understand whether rare mutations in PSD protein-related genes are associated with schizophrenia, we carried out a mutation screening study of 18 PSD protein-related genes in a sample of schizophrenia from Taiwan. Notably, we identified 10 rare missense mutations in the PSD protein-related genes from schizophrenic patients with a family psychiatric history. These 10 missense variants, which were absent from the dbSNP database, the gnomAD (non-neuro) dataset, and Taiwan BioBank, were not detected in the subsequent collection of an independent sample (468 patients with schizophrenia and 533 healthy controls), presuming they might be ultrarare mutations of PSD protein-related genes and exist in the multiplex psychosis family. Our data are also in line with two exome sequencing-based studies that provide strong evidence that *de novo* mutations from schizophrenia affect genes coding for postsynaptic proteins complex (Fromer et al., 2014; Purcell et al., 2014). In addition, similar resequencing studies reported that rare mutations with loss of function in a variety of postsynaptic scaffolding proteins were present in patients with schizophrenia (Xing et al., 2016; Yu et al., 2018).

NLGN2 and NLGN3 are similar in structure and sequence to NLGN1 and share an N-terminal signal peptide, a large esterase homology domain, a single transmembrane region, and a C-terminal cytoplasmic tail (Ichtchenko et al., 1996). We identified three schizophrenia-associated mutants where NLGN2^*R*309*Q*^ and NLGN3^*V*346*M*^ were located at the esterase domain of the NLGN protein, and NLGN3^*Q*691*R*^ was located between the esterase and cholinesterase domain. Notably, one putative damaging missense mutation, NLGN2^*R*309*Q*^, was absent in the 503 controls, 1,517 healthy controls from Taiwan BioBank, the dbSNP database, and the gnomAD (non-neuro) dataset. To understand the effects of NLGN2^*R*309*Q*^, NLGN3^*V*346*M*^, and NLGN3^*Q*691*R*^ on protein expression and translocation, we examined the protein expression and the subcellular localization of the WT and the mutant proteins in HEK-293 and neuro-2a cells. NLGN2 expression were significantly reduced by NLGN2^*R*309*Q*^ in HEK-293 and neuro-2a cells. In addition, NLGN2^*R*309*Q*^ mutant protein was significantly reduced in the membrane fraction of HEK-293 cells and tended to reduce in neuro-2a cells, suggesting that NLGN2^*R*309*Q*^ affects NLGN2 trafficking. Our functional analysis identified the NLGN2^*R*309*Q*^ mutant as a loss-of-function, supporting our previous findings of the association of rare missense mutations in *NLGN2* with schizophrenia (Sun et al., 2011). NLGN3 expression was significantly increased by NLGN3^*R*195*Q*/*V*346*M*^ and reduced by NLGN3^*Q*691*R*^ in HEK-293 cells. However, we did not observe the differential location of NLGN3 protein between NLGN3^*WT*^ and two mutants (NLGN3^*R*195*Q*/*V*346*M*^ and NLGN3^*Q*691*R*^), concluding that the NLGN3 mutants significantly reduced NLGN3 expression but did not affect NLGN3 trafficking. The effect of the NLGN3^*R*195*Q*/*V*346*M*^ and NLGN3^*Q*691*R*^ on schizophrenia pathophysiology requires further elucidation. Taken together, these findings suggest that *NLGN2* and *NLGN3* mutations are part of the genetic underpinnings of schizophrenia and support the implication of synaptic dysfunction in schizophrenia pathogenesis.

SHANK family members share several central domain regions, including a Shank/ProSAP N-terminal domain (SPN), multiple ankyrin (ANK) repeat domains, an SRC homology 3 (SH3) domain, a PSD-05/D/ZO-1 (PDZ) domain, a proline-rich region, and a C-terminal sterile α motif domain (Kursula, 2019). We identified two heterozygous missense mutations (SHANK2^*L*158*F*^ and SHANK3^*P*209*A*^) located at the ANK domain of the SHANK family protein in two unrelated patients with schizophrenia. We speculated that these two mutations might affect the binding ability of SHANK proteins with their interactions with other PSD proteins. Assuming that the SHANK gene mutations affected protein expression, we observed that cells carrying SHANK2^*L*158*F*^ and SHANK3^*P*209*A*^ mutants altered the levels of SHANK2 and SHANK3 proteins, respectively, relative to WT cells. Immunocytochemistry revealed these two mutants and WT proteins mostly localized in the cytosol. Thus, we suggest that these two mutants significantly alter protein levels but do not affect protein trafficking. However, we found that these two SHANK mutant proteins (SHANK2^*L*158*F*^ and SHANK3^*P*209*A*^) were increased in HEK-293 cells but decreased in neuro-2a. Thus, the effect of these mutants on schizophrenia pathophysiology requires further clarification. Peykov and colleagues identified a rare causative SHANK2 variant (SHANK2^*A*1731*S*^) in schizophrenia (Peykov et al., 2015), and several groups have found missense mutations in SHANK3 in schizophrenia (Gauthier et al., 2010; de Sena Cortabitarte et al., 2017). Gauthier and colleagues identified a nonsense mutation of SHANK3 changing an arginine to a stop codon (SHANK3^*R*1117*X*^) in three brothers diagnosed with schizophrenia spectrum disorders (Gauthier et al., 2010). However, increasing evidence is linking SHANK3 gene mutations as a cause of autism (Durand et al., 2007; Waga et al., 2011). Schizophrenia and autism have overlapping symptoms, especially the negative symptoms, suggesting that they share some common biological basis in their pathogenesis. These observations reflect that rare risk mutations in the SHANK gene family might underlie some of the cognitive and social dysfunction present in schizophrenia and autism. Taken together, this suggests that the rare missense variant burden is increased in patients with schizophrenia and those with autism compared with healthy controls.

We previously detected three rare schizophrenia-associated missense mutations (DLGAP3^*G*381*S*^, DLGAP3^*G*587*R*^, and DLGAP3^*R*770*L*^) in patients with schizophrenia (Li et al., 2013a) and speculated that they might affect the posttranslational modification of DLGAP3 protein via kinases phosphorylation. In this study, we identified three additional schizophrenia-associated *DLGAP3* missense mutants (DLGAP3^*H*22*N*^, DLGAP3^*G*352*E*^, DLGAP3^*S*455*R*^) in an independent sample of patients with schizophrenia. Notably, the DLGAP3^*S*455*R*^, DLGAP3^*G*587*R*^, and DLGAP3^*R*770*L*^ mutants were located at the C-terminal of the *DLGAP3* gene, which interacts with focal adhesion kinase and proline-rich tyrosine kinase 2 (Bongiorno-Borbone et al., 2005), presuming the DLGAP3^*S*455*R*^ mutant affects the anchoring of specific protein kinases in the PSD region. We observed that DLGAP3^*H*22*N*^, DLGAP3^*G*352*E*^, and DLGAP3^*S*455*R*^ significantly reduced DLGAP3 protein expression in HEK-293 cells; however, DLGAP3 was expressed in the cytosol in all three mutants and the WT. Thus, we suggest that these mutants significantly reduce DLGAP3 activity but do not affect protein trafficking. Wan and colleagues suggested that DLGAP3 plays a role in inhibiting mGluR5 activity and the downstream triggering of α-amino-3-hydroxy-5-methyl-4-isoxazolepropionic acid receptor endocytosis (Wan et al., 2011). Chen and colleagues identified a role for SAPAP3 in regulating postsynaptic mGluRs and endocannabinoid-mediated synaptic plasticity (Chen et al., 2011). We speculated that DLGAP3^*H*22*N*^, DLGAP3^*G*352*E*^, and DLGAP3^*S*455*R*^ mutants might affect the binding ability of DLGAP3 with proteins associated with metabotropic glutamate receptors. However, these rare *DLGAP3* mutants were not located in a known functional domain but were predicted as highly conserved among different species. Furthermore, we identified a *DLGAP1* missense mutant (DLGAP1^*F*70*L*^) in 1 of 98 patients with schizophrenia, but not in a subsequent collection of 468 patients and 533 healthy controls, suggesting that DLGAP1^*F*70*L*^ might be a patient-specific rare mutation. This missense mutation is located at conserved sequences, presuming that this mutant may impair critical biological functions of DLGAP1. However, the *in silico* analysis and functional analysis by immunoblotting denied this possibility. Thus, the relationship between rare mutations in the DLGAP gene family and schizophrenia pathogenesis needs to be elucidated.

This study has several weaknesses. First, we identified 50 missense mutations in the PSD protein-related genes from our sample. However, we cannot rule out the possibility that common SNPs within these genes may be associated with schizophrenia. Besides, several potentially valuable regions were not sequenced, such as introns and noncoding regions. We also did not detect insertions and deletions variants because of the technical limitation of semiconductor sequencing (Loman et al., 2012). Second, because we were not able to obtain samples from family members of probands, we did not confirm that the identified rare missense mutations were segregated with schizophrenia. Third, our functional assays were conducted using HEK-293 and neuro-2a cells, not primary neuronal cells. Although HEK-293 and neuro-2a cells are straightforward to grow in culture and suitably used as hosts for gene expression, primary neuronal cells are widely used to study synaptic function, morphology, neurotoxicity, neurotransmitter release, and disease modeling. Thus, the findings in the present study must be interpreted cautiously. Fourth, we found that DLGAP3 and DLGAP1 protein were not well detected in neuro-2a cells (data not shown), while the SHANK proteins with the SHANK mutants were increased in HEK-293 cells but decreased in neuro-2a. The discrepant results may be due to cell line–specific expressions and the differences in the genetic and biochemical processes regulating genes or genome activity in human and mouse cells (Zhang et al., 2019). Also, *in vitro* experiments may not fully or accurately predict the effects on a living organism. Further, *in vivo* studies using animal models carrying these mutants should be needed to validate our *in vitro* results and to understand the functional consequence of these mutants *in vivo*. Besides, we were not able to directly evaluate expression changes of PSD-related proteins from patients. Thus, the clinical relevance of the mutations we identified should be interpreted with caution. Finally, a larger sample size is required to have sufficient statistical power to implicate rare pathologic variants with high confidence.

In summary, we identified 10 rare missense mutations in the PSD protein-related genes from schizophrenia, characterized the functional consequences of nine mutations in cultured cells, and demonstrated eight variants altered protein expression and might be associated with schizophrenia. These findings demonstrate the importance of the identified PSD-related gene mutations, especially in the NLGN, SHANK, and DLGAP family, and shed light on future functional genomic investigations of the genes and related biological pathways of schizophrenia. The *in vitro* and *in vivo* impacts of these rare pathological mutations on the pathophysiology of schizophrenia are worthy of future investigation.

## Data Availability Statement

The raw data supporting the conclusions of this article will be made available by the authors, without undue reservation.

## Ethics Statement

The studies involving human participants were reviewed and approved by the Antai Medical Care Cooperation Antai-Tian-Sheng Memorial Hospital Institution Review Board (IRB Number: 101020). The patients/participants provided their written informed consent to participate in this study.

## Author Contributions

M-CC designed the study and wrote the protocol and draft. T-MH, Y-CW, and C-LW helped recruit and evaluate the patients. M-CC, S-HH, and H-YT conducted the experimental works and analyzed the data. All authors reviewed the article and approved its publication.

## Conflict of Interest

The authors declare that the research was conducted in the absence of any commercial or financial relationships that could be construed as a potential conflict of interest.

## Footnotes

1 http://bioinfo.ut.ee/primer3-0.4.0/primer3/

2 https://taiwanview.twbiobank.org.tw

3 http://genetics.bwh.harvard.edu/pph2/

4 http://sift.bii.a-star.edu.sg/

5 http://mmb.pcb.ub.es/PMut/

6 http://provean.jcvi.org/seq_submit.php

7 http://genome.ucsc.edu/index.html

8 http://rsb.info.nih.gov/nih-image/
