# Supplementary material for: Multiple Rare Risk Coding Variants in Postsynaptic Density-Related Genes Associated With Schizophrenia Susceptibility
Source: Front Genet. 2020 Dec 4;11:524258. doi: 10.3389/fgene.2020.524258 (PMC7746813; doi:10.3389/fgene.2020.524258)
Supplement: Supplementary file 1 [file Data_Sheet_1.PDF]

Supplementary Fig. 1

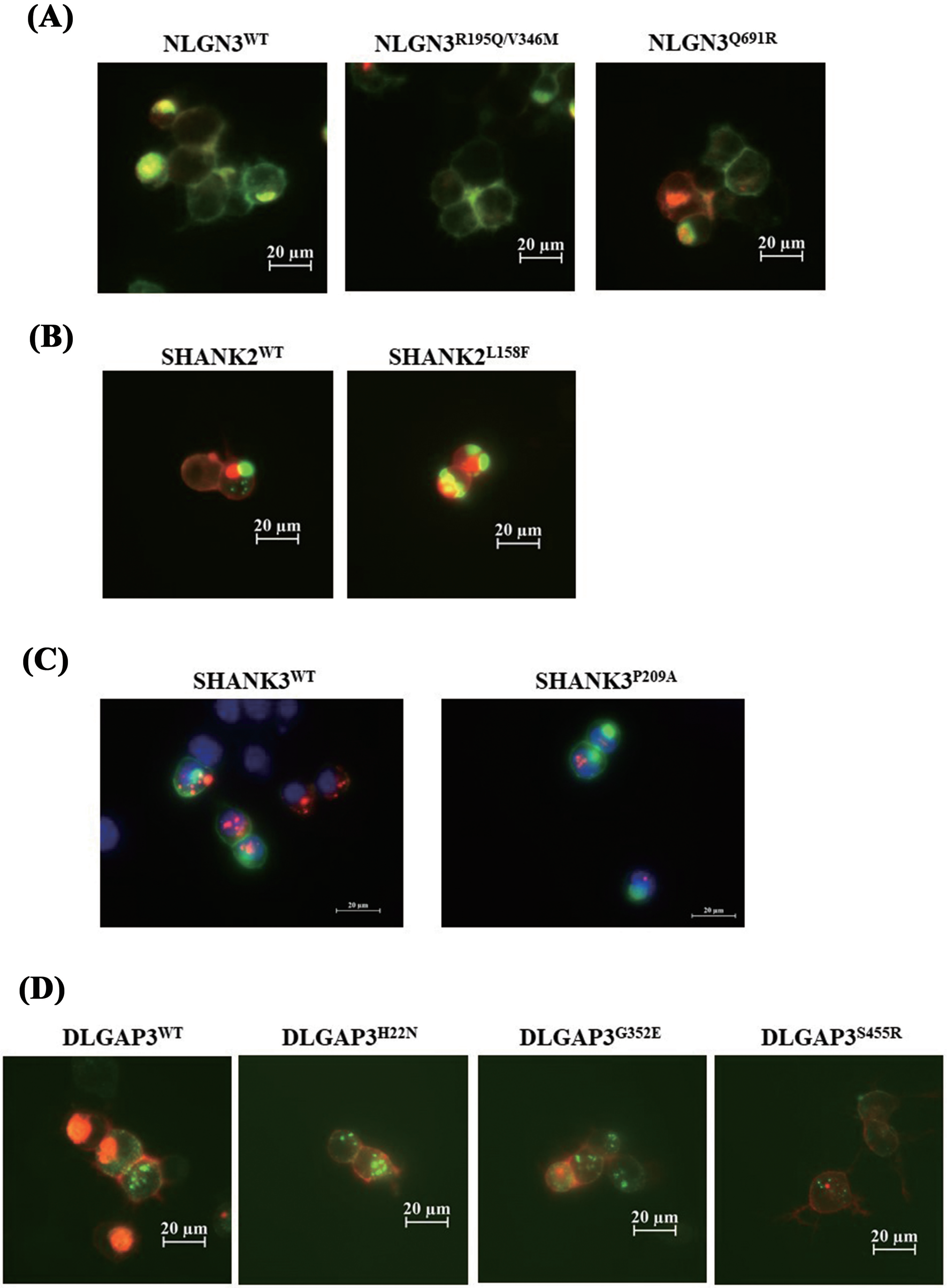

Supplementary Fig. 1. Localization analysis of gene mutants in cultured cells. (A) Subcellular localization of NLGN3 fusion tGFP protein (green) in neuro-2a cells co-transfected with $NLGN3^{WT}$, $NLGN3^{R195Q/V346M}$, or $NLGN3^{Q691R}$ plasmids and LCK plasmids (red). (B) Subcellular localization of SHANK2 fusion tGFP protein (green) in neuro-2a cells co-transfected either with $SHANK2^{WT}$ or $SHANK2^{L158F}$ plasmids and LCK plasmids (red). (C) Subcellular localization of SHANK3 fusion tRFP protein (red) in neuro-2a cells co-transfected either with $SHANK3^{WT}$ or $SHANK2^{P209A}$ plasmids and LCK plasmids (green). Cell nuclei were stained with DAPI (blue). (D) Subcellular localization of DLGAP3 fusion tGFP protein (green) in neuro-2a cells co-transfected with $DLGAP3^{WT}$, $DLGAP3^{H22N}$, $DLGAP3^{G352E}$, or $DLGAP3^{S455R}$ plasmids and LCK plasmids (red). LCK, lymphocyte-specific protein tyrosine kinase membrane protein marker.

Supplementary Table 1. Data generated from the Ion Torrent Personal Genome Machine.

Sample	Output (Mb)	Reads	Mean Read Length (bp)	Average Base Coverage Depth	Variant Calls
PGM1	14.65	111,763	131	100.7	69
PGM2	33.78	262,750	128	227.4	62
PGM3	30.87	246,847	125	207.6	65
PGM4	37.93	301,094	125	254.3	60
PGM5	39.39	315,254	124	261.1	60
PGM6	28.52	223,136	127	188.3	64
PGM7	42.40	330,837	128	282.6	66
PGM8	35.27	291,637	120	223.5	64
PGM9	37.90	301,398	125	252.6	64
PGM10	39.84	315,503	126	258.9	66
PGM11	41.36	314,990	131	283.0	63
PGM12	37.49	285,893	131	259.6	63
PGM13	37.43	293,618	127	254.4	62
PGM14	39.27	311,401	126	264.4	67
PGM15	35.96	288,180	124	241.7	63
PGM16	74,02	509,718	145	513.8	63
PGM17	64,59	444,717	145	447.6	63
PGM18	40,44	280,661	144	282.4	68
PGM19	53,67	372,765	143	370.3	75
PGM20	49,13	337,108	145	336.8	65
PGM21	39,02	280,925	138	270.6	75
PGM22	48,61	336,633	144	335.7	71
PGM23	50,02	348,331	143	346.8	52
PGM24	52,58	369,251	142	367.5	67
PGM25	47,55	326,789	145	330.9	74
PGM26	32,27	223,808	144	225.8	65
PGM27	35,97	248,331	144	254.6	70
PGM28	35,94	254,711	141	251.3	56
PGM29	40,93	279,998	146	287.5	59
PGM30	30,34	219,909	137	213.2	68
PGM31	49	298,349	164	353	82
PGM32	47	290,599	164	343	88
PGM33	41	248,328	168	300	88

PGM34	55	333,120	168	403	86
PGM35	29	173,778	169	213	91
PGM36	27	161,522	169	198	87
PGM37	16	99,775	170	122	87
PGM38	59	356,311	167	431	100
PGM39	52	311,626	168	380	77
PGM40	51	307,558	167	372	88
PGM41	16	98,126	170	121	82
PGM42	45	267,763	168	327	77
PGM43	27	161,335	170	198	83
PGM44	32	194,862	169	237	83
PGM45	34	215,603	161	250	87
PGM46	40	230,502	174	289	79
PGM47	57	340,334	170	421	83
PGM48	16	97,856	172	122	83
PGM49	61	363,856	168	442	96
PGM50	47	281,775	169	344	91
PGM51	44,15	252,832	175	318.8	77
PGM52	22,40	129,742	173	161.6	84
PGM53	81,98	464,734	176	591	76
PGM54	81,28	454,405	179	584.2	79
PGM55	48,35	271,739	178	348.1	79
PGM56	41,17	245,229	168	297.3	74
PGM57	24,49	171,653	143	174.1	87
PGM58	30,92	244,653	126	183.8	89
PGM59	70,07	395,053	177	503.1	67
PGM60	54,02	307,547	176	389.7	91
PGM61	33,84	191,166	177	243.9	83
PGM62	49,69	279,754	178	358.8	82
PGM63	53,34	300,612	177	383.2	74
PGM64	51,50	300,528	171	370.3	81
PGM65	72,75	419,823	173	522.6	73
PGM66	105,57	593,301	178	753.6	71
PGM67	53,85	309,919	174	388.0	86
PGM68	63,37	360,797	176	454.8	92
PGM69	47,43	270,345	175	341.2	80

PGM70	40,48	243,846	166	293.6	91
PGM71	29,23	216,139	135	201.9	83
PGM72	56,19	337,750	166	403.0	81
PGM73	49,77	304,206	164	355.5	66
PGM74	19,87	154,854	128	94.20	90
PGM75	51,10	314,287	163	365.5	82
PGM76	46,30	292,317	158	328.9	89
PGM77	26,52	191,504	139	189.1	78
PGM78	20,33	160,681	127	114.3	85
PGM79	32,41	247,385	131	229.5	68
PGM80	49,84	300,281	166	359.7	90
PGM81	52,98	317,364	167	382.9	88
PGM82	37,69	291,857	129	253.7	91
PGM83	49,70	285,234	174	356.3	86
PGM84	37,82	215,735	175	269.4	73
PGM85	32,10	182,187	176	229.1	80
PGM86	33,14	196,329	169	240.7	88
PGM87	27,64	181,517	152	199.2	91
PGM88	35,60	213,775	167	258.1	81
PGM89	35,27	209,512	168	253.5	90
PGM90	17,69	146,419	121	121.5	92
PGM91	48,85	294,868	166	349.3	89
PGM92	44,04	281,594	156	318.2	84
PGM93	27,40	188,583	145	196.8	80
PGM94	14,51	120,173	121	94.38	79
PGM95	45,76	301,254	152	330.5	78
PGM96	63,62	374,528	170	461.9	82
PGM97	54,85	330,083	166	396.8	83
PGM98	31,45	257,752	122	220.6	71

Supplementary Table 2. Genotypes of 50 missense mutations of the PSD genes in 98 patients with schizophrenia in the semiconductor sequencing stage.

Patient ID or Patient number	*Variant*	*Genotype*			*HWE*
PGM63	DLGAP3_c.64C>A (p.H22N)	*C/C*	*C/A*	*A/A*	0.96
		97	1	0	
PGM96	DLGAP3_c.133_134delCCinsAA (p.P45N)	*GG/GG*	*GG/TT*	*T/T/TT*	0.96
		97	1	0	
PGM13; PGM57	DLGAP3_c.292C>A (p.P98T)	*G/G*	*G/T*	*T/T*	*0.92*
		96	2	0	
PGM83	DLGAP3_c.467C>T (p.T156M)	*C/C*	*C/T*	*T/T*	0.96
		97	1	0	
PGM55	DLGAP3_c.1055G>A (p.G352E)	*G/G*	*G/A*	*A/A*	0.96
		97	1	0	
PGM49	DLGAP3_c.1363A>C (p.S455R)	*A/A*	*A/C*	*C/C*	0.96
		97	1	0	
PGM90	NRXN1_c.3595G>A (p.A1199T)	*C/C*	*C/T*	*T/T*	0.96
		97	1	0	
PGM77	NRXN1_c.3921G>C (p.E1307D)	*C/C*	*C/G*	*G/G*	0.96
		97	1	0	
PGM84	NRXN1_c.3970A>T (p.T1324S)	*T/T*	*T/A*	*A/A*	0.96
		97	1	0	
62 patients	DLGAP2_c.1151C>A (p.P384Q)	*C/C*	*C/A*	*A/A*	0.02

		36	37	25	
PGM62	DLGAP2_c.1516T>C (p.C506R)	*T/T*	*T/C*	*C/C*	0.96
		97	1	0	
PGM77	DLGAP2_c.2290C>G (p.P764A)	*C/C*	*C/T*	*T/T*	0.96
		97	1	0	
PGM90	DLGAP2_c.2750C>T (p.P917L)	*C/C*	*C/T*	*T/T*	0.96
		97	1	0	
30 patients	NRXN2_c.242T>A (p.L81Q)	*A/A*	*A/T*	*T/T*	0.46
		68	26	4	
PGM07; PGM81	NRXN2_c.1442A>T (p.D481V)	*T/T*	*T/A*	*A/A*	0.92
		96	2	0	
PGM83	NRXN2_c.1924C>A (p.P642T)	*C/C*	*C/A*	*A/A*	0.96
		97	1	0	
PGM95	SHANK2_c.8G>A (p.R3H)	*G/G*	*G/A*	*A/A*	0.96
		97	1	0	
PGM72	SHANK2_c.212G>A (p.C71Y)	*G/G*	*G/A*	*A/A*	0.96
		97	1	0	
PGM29; PGM91	SHANK2_c.385G>A (p.V129M)	*C/C*	*C/T*	*T/T*	0.92
		96	2	0	
PGM63	SHANK2_c.431T>C (p.V144A)	*A/A*	*A/G*	*G/G*	0.96
		97	1	0	
PGM80	SHANK2_c.472C>T (p.L158F)	*C/C*	*C/T*	*T/T*	0.96
		97	1	0	

PGM74	SHANK2_c.1237A>G (p.A413T)	*G/G*	*G/A*	*A/A*	0.96
		97	1	0	
PGM19	SHANK2_c.1328G>A (p.R443H)	*G/G*	*G/A*	*A/A*	0.96
		97	1	0	
PGM86; PGM98	SHANK2_c.1604A>G (p.K535R)	*T/T*	*T/C*	*C/C*	0.92
		96	2	0	
PGM47	SHANK2_c.3019G>A (p.A1007T)	*G/G*	*G/A*	*A/A*	0.96
		97	1	0	
PGM16	NRXN3_c.1994G>A (p.R665Q)	*G/G*	*G/A*	*A/A*	0.96
		97	1	0	
PGM85	NRXN3_c.3142G>A (p.G1048S)	*G/G*	*G/A*	*A/A*	0.96
		97	1	0	
PGM50	HOMER2_c.826A>G (p.I276V)	*A/A*	*A/G*	*G/G*	0.96
		97	1	0	
PGM11	NLGN2_c.926G>A (p.R309Q)	*G/G*	*G/A*	*A/A*	0.96
		97	1	0	
PGM61	NLGN2_c.1862G>A (p.R621H)	*G/G*	*G/A*	*A/A*	0.96
		97	1	0	
PGM60	DLGAP1_c.210C>A (p.F70L)	*C/C*	*C/A*	*A/A*	0.96
		97	1	0	
PGM72	DLGAP1_c.1922A>G (p.K641R)	*T/T*	*T/C*	*C/C*	0.96
		97	1	0	
PGM79	SHANK1_c.1267C>G (p.P423A)	*G/G*	*G/C*	*C/C*	0.96

		97	1	0	
18 patients	SHANK1_c.2691C>G (p.D897E)	*G/G*	*G/C*	*C/C*	0.93
		80	17	1	
56 patients	SHANK1_c.4511T>C (p.V1504A)	*A/A*	*A/G*	*G/G*	0.93
		42	44	12	
PGM62	SHANK1_c.5776G>A (p.D1926N)	*C/C*	*C/T*	*T/T*	0.96
		97	1	0	
PGM47; PGM89	SHANK1_c.6203C>G (p.A2068G)	C/C	C/G	G/G	0.92
		96	2	0	
PGM20	SHANK1_c.6220C>T (p.R2074C)	*G/G*	*G/A*	*A/A*	0.96
		97	1	0	
PGM70	DLGAP4_c.1618G>A (p.G540S)	*G/G*	*G/A*	*A/A*	0.96
		97	1	0	
13 patients	DLGAP4_c.2240G>A (p.R747Q)	*G/G*	*G/A*	*A/A*	0.48
		85	*13*	*0*	
PGM10	SHANK3_c.625C>G (p.P209A)	*C/C*	*C/G*	*G/G*	0.96
		97	1	0	
6 patients	SHANK3_c.734T>C (p.I245T)	*T/T*	*T/C*	*C/C*	0.75
		92	6	0	
PGM16; PGM19; PGM67	SHANK3_c.3517G>A (p.A1173T)	*G/G*	*G/A*	*A/A*	0.88
		93	3	0	
PGM27	SHANK3_c.3788C>T (p.P1263L)	*C/C*	*C/T*	*T/T*	0.96
		97	1	0	

PGM94	NLGN3_c.584G>A (p.R195Q)	*G/G*	*G/A*	*A/A*	0.96
		97	1	0	
PGM94	NLGN3_c.1036G>A (p.V346M)	*G/G*	*G/A*	*A/A*	0.96
		97	1	0	
PGM60	NLGN3_c.2072A>G (p.Q691R)	*A/A*	*A/G*	*G/G*	0.96
		97	1	0	
PGM72	NLGN4X_c.71A>G (p.N24S)	*A/A*	*A/G*	*G/G*	0.96
		97	1	0	
PGM80	NLGN4X_c.392A>G (p.N131S)	T/T	T/C	C/C	0.96
		97	1	0	
PGM06	NLGN4Y_c.197T>C (p.I66T)	T/T		C/C	NA
		45		1	

NA= not applicable

Supplementary Table 3. Distribution of 10 rare missense mutations in an independent sample set, including patients with schizophrenia and healthy controls.

Gene symbol	*Variant*	*Amino acid change*	Schizophrenia	Control
DLGAP3	c.64C>A	p.H22N	0/431	0/528
DLGAP3	c.1055G>A	p.G352E	0/409	0/505
DLGAP3	c.1363A>C	p.S455R	0/460	0/529
NRXN2	c.1924C>A	p.P642T	0/449	0/529
SHANK2	c.472C>T	p.L158F	0/405	0/505
NLGN2	c.926G>A	p.R309Q	0/468	0/503
DLGAP1	c.210C>A	p.F70L	0/456	0/533
SHANK3	c.625C>G	p.P209A	0/448	0/526
NLGN3	c.1036G>A	p.V346M	0/461	0/520
NLGN3	c.2072A>G	p.Q691R	0/454	0/531

Supplementary Table 4. Clinical of the eight patients with schizophrenia with rare coding variants identified in this study.

Mutant	*Sex, age (years)*	*Symptoms*	*Family history*	*Patient history*
DLGAP3^{H22N}	Female, 24	Auditory hallucination, persecutory ideation	Mother, schizophrenic; sister, intellectually disabled	Born full term, developmentally delayed in childhood, no history of illicit drug abuse, head injury, or seizure
DLGAP3^{G352E}	Male, 40	Diagnosed at 17 years old, initial psychotic symptoms of self-talking and self-laughing	Brother, schizophrenic	Born full term, unclear developmental history, no history of seizure or head trauma
DLGAP3^{S455R}	Female, 38	Diagnosed at ± 20 years old, auditory hallucination and bizarre behavior	Mother, schizophrenic	Insignificant developmental history
SHANK2^{L158F}	Female, 50	initial symptoms of bizarre behavior (such as defecating in the room),	Sister and cousins schizophrenic	Insignificant birth and developmental history

		developing delusions of being poisoned		
NLGN2^{R309Q}	Female, 41	Initial psychotic symptoms of auditory hallucination and persecutory delusion, current symptoms of olfactory hallucination, mannerisms, self-laughing behavior, deteriorating cognitive function, and poor self-care	Mother and sister, schizophrenic	Premature birth, insignificant developmental history
DLGAP1$^{F70L/}$NLGN3^{Q691R}	Female, 37	Diagnosed at the age of 19 years, initial psychotic symptoms of auditory hallucination and bizarre behavior	Brother, schizophrenic	
SHANK3^{P209A}	Female, 48	Initial psychotic symptoms of an auditory hallucination and bizarre delusion	Mother, schizophrenic; daughter and husband, intellectually disabled	History of alcohol abuse
NLGN3$^{R195Q/V346M}$	Male, 54	Diagnosed at 19 years old, initial	Brother, intellectually	Insignificant birth and

		symptoms of reference delusions followed by prominent erotic delusions, ideas of reference, and thought broadcasting	disabled.	developmental history
